# Xuebijing injection combined with alprostadil in the treatment of diabetic nephropathy: A PRISMA-compliant systematic review and meta-analysis

**DOI:** 10.1097/MD.0000000000032095

**Published:** 2024-06-14

**Authors:** Na Hao, Yang Liu, LuXuan Guo, Wanwen Li, Pengbo Zhao

**Affiliations:** a First Teaching Hospital of Tianjin University of Traditional Chinese Medicine, Tianjin, China; b National Clinical Research Center for Chinese Medicine Acupuncture and Moxibustion, Tianjin, China.

**Keywords:** alprostadil, diabetic nephropathies, meta-analysis, randomized controlled trials, Xuebijing injection

## Abstract

**Background:**

Diabetes nephropathy (DN), as one of the common complications of diabetes, is characterized by persistent albuminuria, decreased glomerular filtration rate, and elevated arterial blood pressure. At present, Xuebijing injection is widely used in the treatment of DN. However, few systematic reviews and meta-analysis related to Xuebijing injection intervention in DN were published. In order to more systematically and objectively evaluate the clinical efficacy of Xuebijing injection intervention in DN, we conducted systematic reviews and meta-analysis to verify it.

**Objective:**

The purpose of the research was to systematically evaluate the clinical efficacy of Xuebijing injection combined with alprostadil in the treatment of diabetic nephropathy.

**Methods:**

We searched the China National Knowledge Infrastructure (CNKI), China Biomedical Database (SinoMed), Weipu Database (VIP), Wanfang Database, PubMed, The Cochrane Library, Embase, Web of Science and other databases by computer, and searched the randomized controlled trials of Xuebijing injection combined with alprostadil in the treatment of DN at home and abroad from the establishment of the database to 2022. The main outcome indicators included blood glucose, and the secondary outcome indicators included blood lipid, renal function, urinary protein, and safety. Two evaluators independently screened the literature, extracted the data and evaluated the risk of bias in the included studies. RevMan 5.3 software was used to analyze the data.

**Results:**

A total of 14 randomized controlled trials were included, including 1233 cases, 618 cases in the treatment group and 615 cases in the control group. The results of meta-analysis demonstrated that compared with the control group, the treatment group could effectively reduce fasting plasma glucose [mean difference [MD] = −1.90, 95% CI (−2.40, −1.40), *P* < .00001], glycosylated hemoglobin A1c [MD = −2.38, 95% CI (−2.51, −2.25), *P* < .00001], 2h postprandial blood glucose [MD = −2.92, 95% CI (−3.95, −1.89), *P* < .00001], triacylglycerol [MD = −1.08, 95% CI (−1.66, −0.50), *P* = .0003], total cholesterol [MD = −1.17, 95% CI (−1.39, −0.95), *P* < .00001], low-density lipoprotein cholesterol [MD = −1.19, 95% CI (−1.60, −0.78), *P* < .00001], high-density lipoprotein cholesterol [MD = 0.32, 95% CI (0.23, 0.42), *P* < .00001], serum creatinine [MD = −42.95, 95% CI (−57.46, −28.43), *P* < .00001], blood urea nitrogen [MD = −2.24, 95%CI (−2.62,−1.86), *P* < .00001], blood β2 microglobulin [SMD = −1.49, 95% CI (−1.70, −1.28), *P* < .00001], urine β2 microglobulin [SMD = −0.81, 95% CI (−1.04, −0.58), *P* < .00001], 24-hour urinary protein quantification [MD = −0.20, 95% CI (−0.26, −0.14), *P* < .00001], urinary albumin excretion rate [SMD = −1.15, 95% CI (−1.38, −0.93), *P* < .00001].

**Conclusion:**

Xuebijing injection combined with alprostadil has more advantages in treating DN compared to routine Western medicine.

## 1. Introduction

Diabetes nephropathy (DN) is one of the common complications of diabetes. It is characterized by persistent albuminuria, reduced glomerular filtration rate and elevated arterial blood pressure.^[1]^ In recent years, the number of patients with diabetes has expanded. In 2021, the prevalence rate of diabetes among people aged 20 to 79 in the world was 10.5% (536.6 million), and it is anticipated to increase to 12.2% in 2045.^[2]^ The epidemiological study on DN in China shows that the prevalence of DN in the diabetes population is about 20% to 40%.^[3]^ The treatment of DN mainly predominantly on controlling blood glucose and blood pressure, combined with adjusting lifestyle, quitting smoking and limiting protein intake, which can delay the progress of DN.^[4]^ In recent years, Traditional Chinese Medicine has gained good curative effects in reducing DN symptoms, assisting in stable blood pressure reduction, glucose and lipid reduction, strengthening renal function, and delaying the progress of DN.^[5,6]^ Xuebijing injection (XBJI) is a kind of traditional Chinese medicine injection, which is extracted from the effective components of traditional Chinese medicine such as Honghua, Chishao, Chuanxiong, Danshen, Danggui. It has the effects of promoting blood circulation and removing blood stasis, clearing heat and detoxification, strengthening health and tonifying deficiency.^[7]^ Modern research has identified that it has the effects of enhancing microcirculation, inhibiting the accumulation of inflammatory mediators, anti-inflammatory and anti-oxidation. It is generally used in various system infections and toxic diseases.^[8]^ In recent years, clinical trials of XBJI in the treatment of DN have progressively increased.^[9–11]^ However, due to different outcome indicators, inconsistent research quality and small sample size in most studies, it is not viable to provide reliable guidance for clinical practice. Accordingly, author gathered the clinical trials of XBJI combined with alprostadil in the treatment of DN and conducted meta-analysis, so as to provide a reliable evidence based basis for the clinical application of XBJI in DN, and at the same time provide a certain reference value for the further extensive use of Xuebijing injection in the clinical intervention of DN and the delay of the progress of kidney disease.

## 2. Methods

### 2.1. Search strategy

Chinese search terms included“糖尿病肾病”“糖尿病性肾小球硬化症”“糖尿病性肾病”“糖尿病肾损伤”“糖尿病肾脏病”“血必净注射液”“血必净”and so on. English search terms were as follows: “Diabetic Nephropathies” “Diabetic glomerulosclerosis” “Diabetic nephropathy” “Diabetic renal injury” “Diabetic kidney disease” “Xuebijing injection” “Xuebijing,” etc. Subject words and free words form the retrieval type of database search. All randomized controlled trials of XBJI combined with modern medicine in the treatment of DN were collected. The search strategies take CNKI and PubMed as examples (Table 1).

**Table 1:** Search strategy.

	CNKI	Pubmed
#1	糖尿病肾病	Diabetic nephropathies [Mesh]
#2	糖尿病性肾小球硬化症	Diabetic glomerulosclerosis [Title/Abstract]
#3	糖尿病性肾病	Diabetic nephropathy [Title/Abstract] Diabetes nephropathy
#4	糖尿病肾损伤	Diabetic renal injury [Title/Abstract]
#5	糖尿病肾脏病	Diabetic kidney disease [Title/Abstract]
#6	#1 OR #2 OR #3 OR #4 OR #5	
#7	血必净射液	Xuebijing injection [Title/Abstract]
#8	血必净	Xuebijing [Title/Abstract]
#9	#7 OR #8	
#10	#6 AND #9	

### 2.2. Inclusion criteria

1. Study type: Randomized controlled trials (RCTs) of XBJI combined with alprostadil in the treatment of DN.
2. Participants: DN was definitely diagnosed by Western medicine, without serious abnormal heart, liver and kidney function, and the patient nationality, age and gender were unlimited.
3. Intervention measures: The incorporated population were given basic treatments such as lifestyle intervention, diet control, hypoglycemic, antihypertensive and lipid-lowering. On this basis, the control group was treated with alprostadil and the treatment group was treated with XBJI on the basis of the control group.
4. Outcome indicators: main outcome measures: blood glucose index: fasting plasma glucose (FPG), glycosylated hemoglobin A1c (HbA1c), 2h postprandial blood glucose (2hPG); Secondary outcome measures: blood lipid indexes: triacylglycerol (TG), total cholesterol (TC), low-density lipoprotein cholesterol (LDL-C), high-density lipoprotein cholesterol (HDL-C); renal function indexes: serum creatinine (Scr), blood urea nitrogen (BUN), blood β2 microglobulin, urine β2 microglobulin; urinary protein index: 24-hour urinary protein quantification (24hUp), urinary albumin excretion rate (UAER); (iv)safety index: Adverse reactions

### 2.3. Exclusion criteria

If the above conditions are not satisfied, the literature will be excluded. In addition, research that meet the following conditions should also be excluded:

1. Does not satisfy the DN diagnostic criteria.
2. Repeatedly published papers.
3. Review, case reports, animal experiments and other non-RCTs literature.
4. The intervention measures of treatment group or control group combined with other traditional Chinese medicine treatment.
5. Studies with incomplete data and unable to contact the author for complete data.

### 2.4. Study selection and data extraction

Two evaluators (NH and YL) independently screened the literature according to the inclusion and exclusion criteria and cross-checked the inclusion results. If there were obvious errors or vague information in the research, we could contact the author of the studies by e-mail. If valid original data could not be obtained, we could consider abandoning the studies with problems. In case of disagreement, the 2 evaluators should discuss and determine with the third researcher (Luxuan Guo). Excel 2013 was used to extract the data from the involved literature. The extracted contents include: (clinical research (title, first author, date of publication, sample size, average course of disease); Intervention measures (treatment frequency and course of treatment of intervention scheme in control group and treatment group); Outcome indicators.

### 2.5. Literature quality evaluation

Two evaluators (NH and YL) assessed the literature quality of the incorporated research according to the Cochrane manual, including the generation of random sequences, allocation concealment, blind measures, the integrity of outcome indicators, selective reports, and other biases.

### 2.6. Statistical analysis

Revman 5.3 software was used for analysis. The relative risk and mean difference (MD) were used as efficacy indicators for counting data and continuity variables. If the numerical units were inconsistent, the standardized mean difference (SMD) was used for analysis. The Chi-square test was used to test the heterogeneity of results. If *P* > .10, I^2^ < 50%, it was considered that the heterogeneity among studies was small, and the fixed effect model was used. If *P ≤ *.10, I^2^ ≥ 50%, it was considered that there was great heterogeneity among studies, and the random effect model was used, and the source of heterogeneity should be further evaluated through subgroup analysis or sensitivity analysis. If combined analysis was not viable, descriptive analysis should be utilized. Inverted funnel plots were used to determine potential publication bias when more than 10 studies were included in the meta-analysis.

## 3. Results

### 3.1. Literature search results

**Figure 1.**
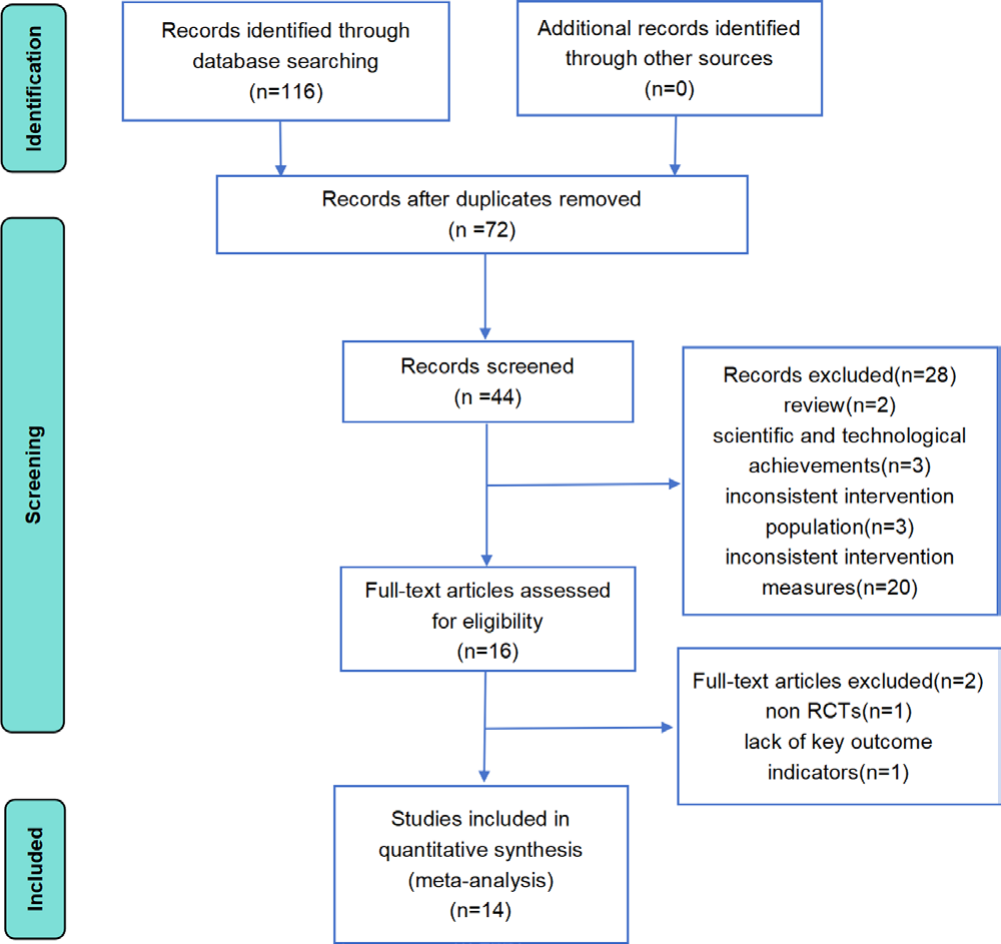
116 researchers were initially retrieved, including CNKI (31), Wanfang (37), VIP (24), SinoMed (24). After screening, 14 RCTs meeting the inclusion and exclusion criteria were finally included.

116 researchers were initially retrieved, including CNKI (31), Wanfang (37), VIP (24), SinoMed (24). After eliminating the duplicate literature, the remaining 44 studies were screened by reading the titles and abstracts, and the reviews (2), scientific and technological achievements (3), inconsistent intervention population (3) and inconsistent intervention measures (20) were excluded. The remaining 16 studies were rescreened, excluding non-RCT (1) and missing key outcome indicators (1). Ultimately, 14 RCTs^[9–22]^ were included, with a total of 1233 patients (Fig. 1).

### 3.2. Study characteristics

The involved people were all Chinese, and the test sites were all in China. A total of 14 RCTs were included, including 1233 cases, 618 cases in the treatment group and 615 cases in the control group. Sample sizes for individual studies ranged from 29 to 156 people, and the therapy course was 1 to 4 weeks. The treatment group was treated with XBJI combined with alprostadil, and the control group was treated with alprostadil. Both groups were given basic treatment such as lifestyle intervention, diet control, hypoglycemic and lipid-lowering (Table 2).

**Table 2:** Study characteristics.

Study	Sample size		Average course of DN (yr/mo)		Intervention		Treatment cycle	Outcome
	T	C	T	C	T	C		
Yu N 2021^[8]^	42	42	8.22 ± 1.27 (Y)	8.23 ± 1.28 (Y)	XBJI (50 mL) + Alprostadil (10 μg), iv drip, once/d	Alprostadil (10 μg), iv drip, once/d	4 wk	⑧⑨⑫
Liu D Z 2021^[9]^	76	76	NR	NR	XBJI (50 mL) + Alprostadil (10 μg), iv drip, once/d	Alprostadil (10 μg), iv drip, once/d	4 wk	⑧⑨
Zeng R B 2019^[10]^	41	41	5.78 ± 1.31 (Y)	5.22 ± 1.21 (Y)	XBJI (10 mL) + Alprostadil (2 mL), iv drip, once/d	Alprostadil (2 mL), iv drip, once/d	2 wk	⑧⑨⑫
Yu P 2019^[11]^	40	40	NR	NR	XBJI (50 mL) + Alprostadil (10 μg), iv drip, once/d	Alprostadil (10 μg), iv drip, once/d	4 wk	④⑤⑥⑦⑧⑨⑫
Cai F L 2019^[12]^	31	31	2.0 ± 0.4 (Y)	2.1 ± 0.3 (Y)	XBJI (20 mL) + Alprostadil (20 μg), iv drip, once/d	Alprostadil (20 μg), iv drip, once/d	2 wk	⑭
Zhang Y L 2019^[13]^	40	40	NR	NR	XBJI (40 mL) + Alprostadil (10 μg), iv drip, once/d	Alprostadil (10 μg), iv drip, once/d	1 wk	⑧⑨⑫
Liu Q L 2018^[14]^	31	31	11.32 ± 3.12 (M)	11.65 ± 4.24 (M)	XBJI (50 mL) + Alprostadil (20 μg), iv drip, 2 times/d	Alprostadil (20 μg), iv drip,2 times/d	2 wk	⑧⑨⑩⑪
Dong J Y 2018^[15]^	50	50	1.02 ± 0.45 (Y)	0.98 ± 0.45 (Y)	XBJI (50 mL) + Alprostadil, iv drip, once/d	Alprostadil, iv drip, once/d	4 wk	①②③⑧⑨⑩⑪
Jiang Q 2017^[16]^	78	78	6.02 ± 2.2 (Y)	6.11 ± 2.44 (Y)	XBJI (50 mL) + Alprostadil (20 μg), iv drip, 2 times/d	Alprostadil (20 μg), iv drip,2 times/d	2 wk	①②④⑤⑥⑦⑧⑨⑫⑬
Chen K S 2017^[17]^	45	45	10.06 ± 5.17 (M)	9.52 ± 5.75 (M)	XBJI (50 mL) + Alprostadil (10 μg), iv drip, once/d	Alprostadil (10 μg), iv drip, once/d	4 wk	①③② ④⑤⑥⑦⑧⑨⑩⑪
Dong X 2017^[18]^	66	66	NR	NR	XBJI (10 mL) + Alprostadil (2 mL), iv drip, once/d	Alprostadil (2 mL), iv drip, once/d	2 wk	①②⑤⑥⑦⑧⑨⑩⑫⑬
Cao L 2016^[19]^	33	31	NR	NR	XBJI (10 mL) + Alprostadil (2 ml), iv drip, once/d	Alprostadil (2 mL), iv drip, once/d	2 wk	①②④ ⑤⑥⑦⑧⑨⑩⑪⑫⑬
Li Q 2016^[20]^	30	30	5.9 ± 2.6 (Y)	5.6 ± 2.7 (Y)	XBJI (40 mL) + Alprostadil (10 μg), iv drip, once/d	Alprostadil (10 μg), iv drip, once/d	1 wk	⑧⑨⑫
Wang Q Y 2014^[21]^	15	14	10.05 ± 2.55 (Y)	10.74 ± 3.35 (Y)	XBJI (100 mL) + Alprostadil (10 μg), iv drip, once/d	Alprostadil (10 μg), iv drip, once/d	2 wk	⑫

C = Control group; NR = Not reported, T = Treatment group.

Blood glucose index: ① FPG; ② 2hPG; ③ HbA1c.

Blood lipid index: ④ TG; ⑤ TC; ⑥ LDL-C; ⑦ HDL-C.

Renal function indexes: ⑧ Scr; ⑨ BUN; ⑩ blood β2 microglobulin; ⑪ urine β2 microglobulin.

Urinary protein index: ⑫ 24hUp; ⑬ UAER. safety index.

Safety index: ⑭adverse reactions.

### 3.3. Assessment of risk of bias

**Figure 2.**
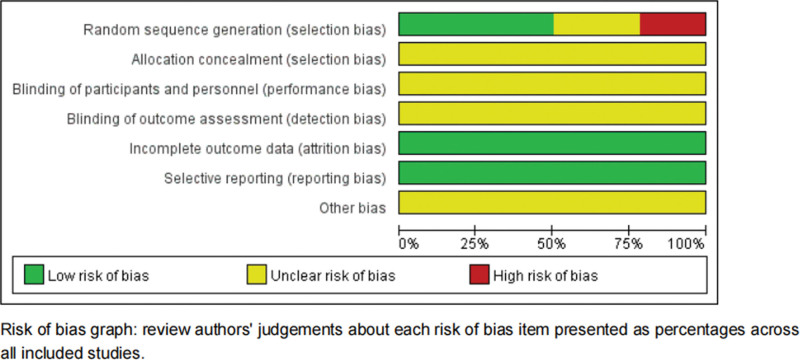
The Cochrane Bias Risk Assessment tool was used to assess the risk of bias in the 14 included studies.

The Cochrane Bias Risk Assessment tool was used to assess the risk of bias in the 14 included studies. Six studies^[9,11,13,15,18,19]^ were evaluated as “low risk” by random number table, 1 study^[22]^ was evaluated as “low risk” by lottery method, 3 studies^[10,14,17]^ were grouped according to different treatment methods and evaluated as “high risk,” and the other 4 studies^[12,16,20,21]^ did not specify the random method and evaluated as “unclear risk.” None of the incorporated studies mentioned allocation concealment and blinding and were evaluated as “unclear risk.” All studies^[9–22]^ had no loss of follow-up and withdrawal and we evaluated as “low risk.” Fourteen studies were^[9–22]^ not found to involve selective reports, which were evaluated as “low risk.” Other biases were unknown and were evaluated as “unclear risk” (Fig. 2).

### 3.4. Meta-analysis results of the main outcome indicators

#### 3.4.1. Meta-analysis of blood glucose index

1. Fasting plasma glucose AND 2h postprandial blood glucose

Five studies^[12,13,18,19,22]^ contained indicators related to FPG, with 272 cases in the treatment group and 270 cases in the control group. The random effect model was adopted due to high heterogeneity (*P* < .00001, I^2^ = 89%), the results showed that the difference between the treatment group and the control group in reducing the level of FPG was statistically significant [MD = −1.90, 95%CI (−2.40, −1.40), *P* < .00001]. The heterogeneity still existed after subgroup analysis according to the treatment course difference between the groups and the dosage of XBJI. Consequently, sensitivity analysis was carried out to exclude each research one-by-one. When Jiang Qiang^[13]^ was excluded, the heterogeneity changed markedly (*P* = .80, I^2^ = 0%). The fixed effect model was adopted, meta-analysis results showed that the difference between the treatment group and the control group was statistically significant [MD = −2.07, 95% CI (−2.27, −1.87), *P* < .00001].

**Figure 3.**
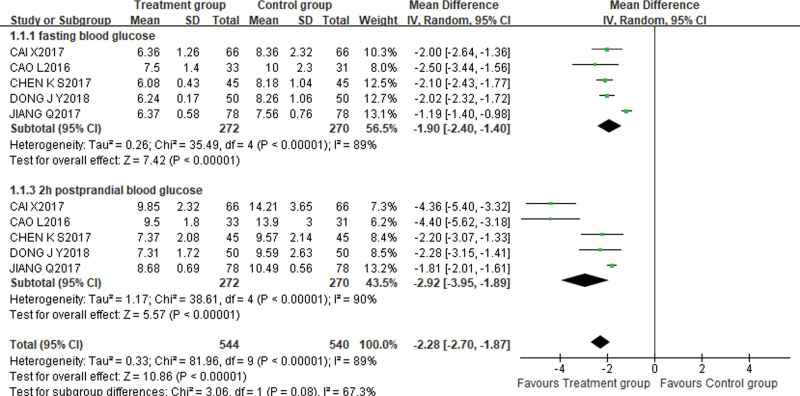
On the comparison of fasting blood glucose indicators, the difference between the control group and the treatment group was statistically significant [MD = −1.90, 95%CI (−2.40, −1.40), *P* < .00001]. At the same time, the meta-analysis results demonstrated that compared with the control group, the treatment group had significant advantages in lowering the level of 2h postprandial blood glucose [MD = −2.92, 95%CI (−3.95, −1.89), *P* < .00001].

Five studies^[12,13,18,19,22]^ incorporated indicators related to 2hPG, with 272 cases in the treatment group and 270 cases in the control group. The data were evaluated using the random effects model due to the high heterogeneity (*P* < .00001, I^2^ = 90%), and the results of the meta-analysis showed that the treatment group was significantly better than the control group in reducing the level of 2hPG [MD = −2.92, 95%CI (−3.95, −1.89), *P* < .00001]. The heterogeneity was still large after subgroup analysis according to the difference in treatment course between groups. After subgroup analysis according to the dosage specification of XBJI, the heterogeneity was significantly reduced in both 10 mL^[18,19]^(*P* = .96, I^2^ = 0%) and 50mL^[12,13,22]^ (*P* = .42, I^2^ = 0%) XBJI groups. Meta-analysis results demonstrated that compared with the control group, the treatment group had significant advantages in lowering the level of 2hPG [MD_10mL_ = −4.38, 95% CI (−5.17, −3.58), *P* < .00001], [MD_50mL_ = −1.85, 95% CI (−2.04, −1.66), *P* < .00001] (Fig. 3).

2. Glycosylated hemoglobin A1c

**Figure 4.**
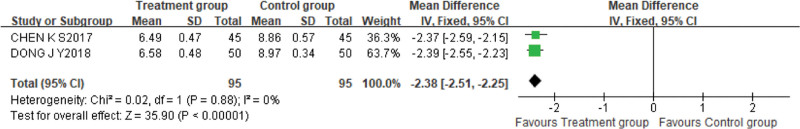
The meta-analysis demonstrated that the treatment group was superior to the control group in reducing the level of glycosylated hemoglobin A1c [MD = −2.38, 95% CI (−2.51, −2.25), *P* < .00001].

Two studies^[12,22]^ included indicators related to HbA1c, with 95 cases in the treatment group and 95 cases in the control group. The fixed effect models were used due to lower heterogeneity (*P* = .88, I^2^ = 0%). Meta-analysis demonstrated that the treatment group was superior to the control group in reducing the level of HbA1c [MD = −2.38, 95% CI (−2.51, −2.25), *P* < .00001] (Fig. 4).

### 3.5. Meta-analysis results of the secondary outcome indicators

#### 3.5.1. Meta-analysis of blood lipid indexes

1. Triacylglycerol, total cholesterol and low-density lipoprotein

Four incorporated studies^[13,17,19,22]^ used TG as an outcome indicator, with 196 cases in the treatment group and 194 cases in the control group. The random effect model was adopted due to high heterogeneity (*P* < .00001, I^2^ = 98%). Meta-analysis demonstrated that the treatment group was superior to the control group in reducing the level of TG [MD = −1.08, 95% CI (−1.66,−0.50), *P* = .0003]. Subgroup analysis was carried out according to the difference of treatment course between groups to explore the source of heterogeneity. The heterogeneity of 4-week group^[17,22]^ was considerably reduced (*P* = .90, *I^2^* = 0%). The results of meta-analysis confirmed the superiority of the treatment group [MD = −1.62, 95% CI (−1.77, −1.47), *P* = .0003]. The heterogeneity of 2-week group^[13,19]^ was still very high (*P* = .0006, *I^2^* = 91%), but meta-analysis also confirmed that the treatment group was superior to the control group in reducing the level of TG [MD = −0.54, 95% CI (−0.85, −0.24), *P* = .0004]. Further heterogeneity analysis of 2-week group discovered that Jiang Qiang^[13]^ used 50mL XBJI and Cao Li^[19]^ used 10mL XBJI, maybe the difference in XBJI dose was the source of heterogeneity.

Five studies^[13,17–19,22]^ used TC as an outcome indicator, with 262 cases in the treatment group and 260 cases in the control group. The random effect model was adopted due to high heterogeneity (*P* < .00001, I^2^ = 92%). The results of meta-analysis confirmed the superiority of the treatment group [MD = −1.17, 95%CI (−1.39, −0.95), *P* < .00001]. Subgroup analysis was performed to determine the source of heterogeneity according to the difference in treatment course between groups. The heterogeneity of 2-week group^[13,18,19]^ and 4-week group^[17,22]^ decreased significantly [2-week group (*P* = .68, *I^2^* = 0%), 4-week group (*P* = .68, I^2^ = 0%)]. The meta-analysis results of the 2 subgroups certified that the treatment group was better than the control group in lowering the level of TC [MD_2-week group_ = −1.34, 95%CI (−1.43, −1.25), *P* < .00001], [MD_4-week group_ = −0.91, 95%CI (−0.98, −0.84), *P* < .00001]. It is suggested that the heterogeneity between groups may be related to the therapy cycle.

**Figure 5.**
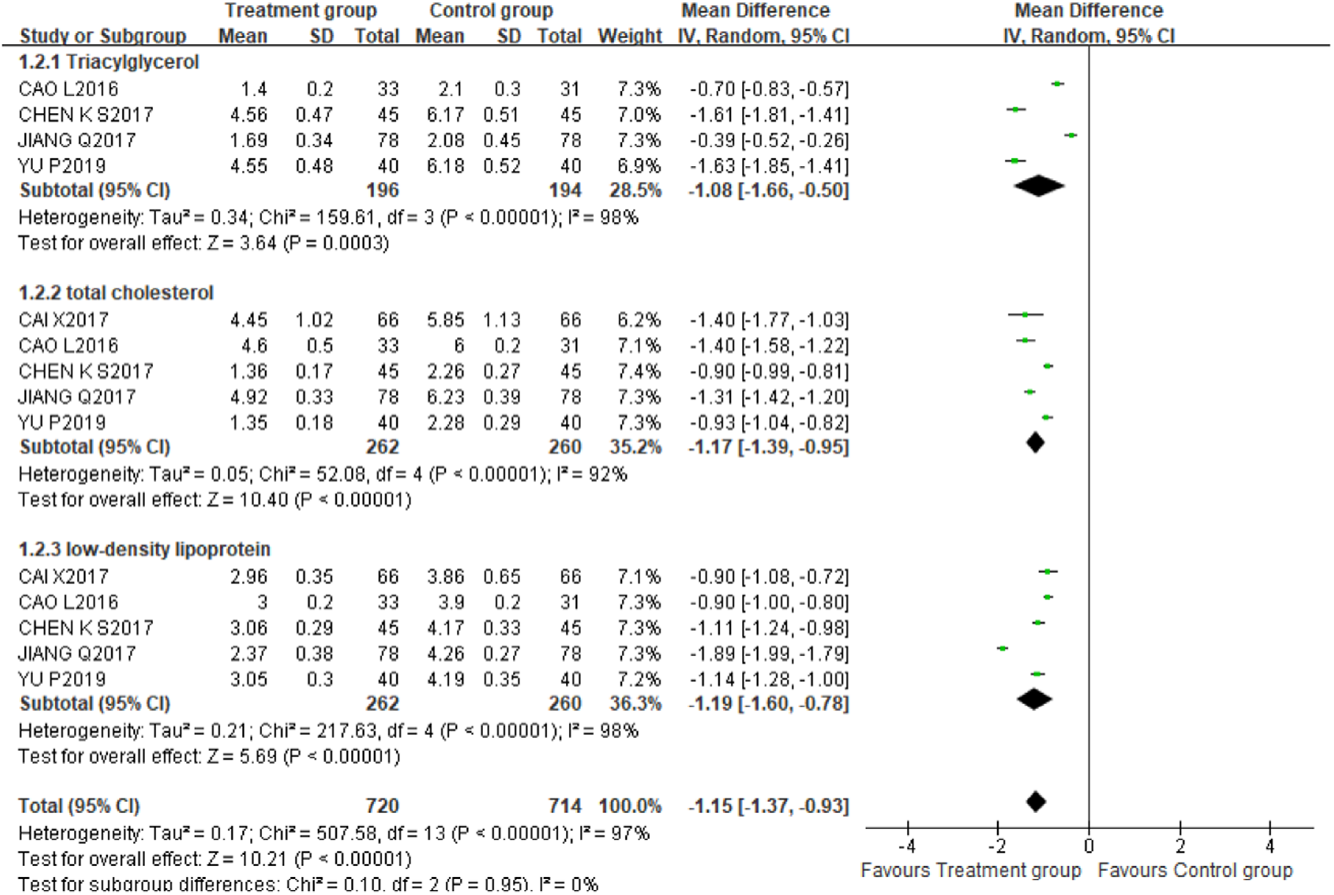
On the comparison of triacylglycerol indicators, the difference between the control group and the treatment group was statistically significant [MD = −1.08, 95% CI (−1.66,−0.50), *P* = .0003]. In terms of the indexes affecting total cholesterol, the difference between the treatment group and the control group was statistically significant. [MD = −1.17, 95%CI (−1.39, −0.95), *P* < .00001]. The treatment group is superior to the control group in reducing low-density lipoprotein [MD = −1.19, 95%CI (−1.60, −0.78), *P* < .00001].

Five studies^[13,17–19,22]^ used LDL-C as an outcome indicator, with 262 cases in the treatment group and 260 cases in the control group. The random effect model was adopted due to high heterogeneity (*P* < .00001, I^2^ = 98%). The results of the meta-analysis confirmed the superiority of the treatment group [MD = −1.19, 95%CI (−1.60, −0.78), *P* < .00001] (Fig. 5).

2. High-density lipoprotein

**Figure 6.**
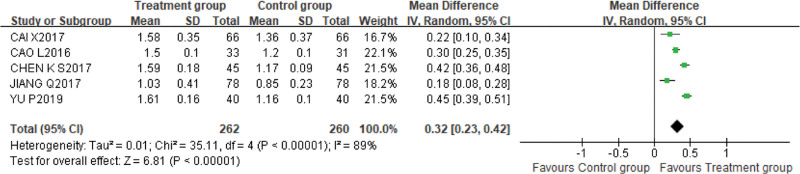
The results of meta-analysis showed that the combination of alprostadil and XBJI could better improve the level of high-density lipoprotein [MD = 0.32, 95%CI (0.23, 0.42), *P* < .00001].

Five studies^[13,17–19,22]^ used HDL-C as an outcome indicator, with 262 cases in the treatment group and 260 cases in the control group. The random effect model was adopted due to high heterogeneity (*P* < .00001, I^2^ = 89%). The results of meta-analysis showed that the combination of alprostadil and XBJI could better improve the level of HDL-C [MD = 0.32, 95%CI (0.23, 0.42), *P* < .00001] (Fig. 6).

#### 3.5.2. Meta-analysis of renal function indexes

1. Serum creatinine

**Figure 7.**
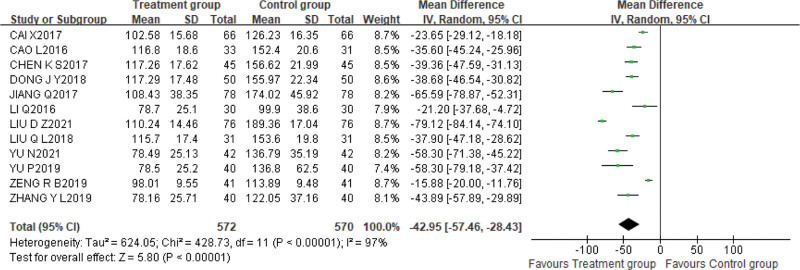
The results of meta-analysis showed that the combination of alprostadil and XBJI could better decrease the level of serum creatinine [MD = −42.95, 95%CI (−57.46, −28.43), *P* < .00001].

A total of 12 studies^[9,10,12–19,21,22]^ reported the levels of Scr after treatment, with 572 cases in the treatment group and 570 cases in the control group. The random effect model was adopted due to high heterogeneity (*P* < .00001, I^2^ = 97%). The results of meta-analysis showed that the combination of alprostadil and XBJI could better decrease the level of Scr [MD = −42.95, 95%CI (−57.46, −28.43), *P* < .00001]. In order to explore the source of the heterogeneity, according to the XBJI dosage and course of treatment after subgroup analysis still existed large heterogeneity. Therefore, we used the one-by-one elimination method to analyze the source of heterogeneity. When the heterogeneity did not change significantly after excluding any studies, in addition, the confidence intervals in the forest map were all located on the left side of the invalid line, indicating that the heterogeneity between studies did not affect the results (Fig. 7).

2. Blood Urea nitrogen

**Figure 8.**
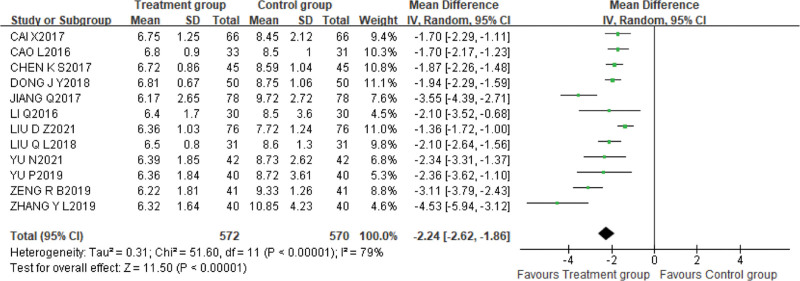
The results of the meta-analysis showed that the treatment group was significantly better than the control group in reducing the level of blood urea nitrogen [MD = −2.24, 95%CI (−2.62, −1.86), *P* < .00001].

Twelve studies^[9,10,12–19,21,22]^ used BUN as an outcome indicator, with 572 cases in the treatment group and 570 cases in the control group. The data were evaluated using the random effects model due to the high heterogeneity (*P* < .00001, I^2^ = 79%), and the results of meta-analysis showed that the treatment group was significantly better than the control group in reducing the level of BUN [MD = −2.24, 95%CI (−2.62, −1.86), *P* < .00001]. Subgroup analysis based on dose and duration of XBJI still showed considerable heterogeneity. Therefore, Sensitivity analysis was used to explore the source of heterogeneity, when any studies were excluded, heterogeneity did not modify substantially, and the confidence intervals in the forest map were all on the left side of the invalid line, demonstrating that heterogeneity between studies did not alter the results (Fig. 8).

3. Blood β2 microglobulin and Urine β2 microglobulin

A total of 5 studies^[12,16,18,19,22]^ reported the levels of blood β2 microglobulin, with 225 cases in the treatment group and 223 cases in the control group. Due to the inconsistency of units between studies, SMD was used for analysis. The fixed effect models were used due to lower heterogeneity (*P* = .63, *I^2^* = 0%). Meta-analysis demonstrated that the treatment group was superior to the control group in reducing the level of blood β2 microglobulin [SMD = −1.49, 95%CI (−1.70, −1.28), *P* < .00001].

**Figure 9.**
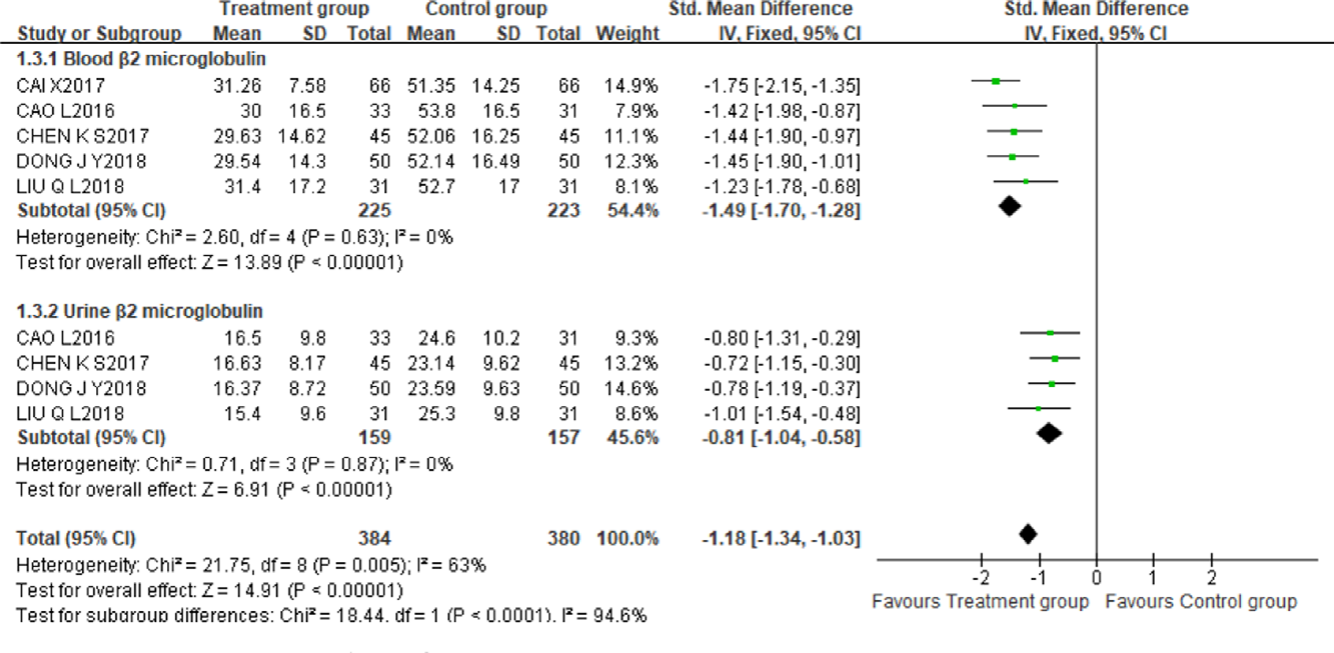
Meta-analysis demonstrated that the treatment group was superior to the control group in reducing the level of bloodβ2 microglobulin [SMD = −1.49, 95%CI (−1.70, −1.28), *P* < .00001]. On the comparison of the outcome indicators of Urineβ2 microglobulin, the results of meta-analysis verified that the efficacy of XBJI with alprostadil was better than that of alprostadil alone [SMD = −0.81, 95%CI (−1.04, −0.58), *P* < .00001].

Four studies^[12,16,19,22]^ used urine β2 microglobulin as an outcome measure, with 159 cases in the treatment group and 157 cases in the control group. Due to the inconsistency of units between studies, SMD was used for analysis. The fixed effect models were used due to lower heterogeneity (*P* = .87, *I^2^* = 0%). The results of meta-analysis verified that the efficacy of XBJI with alprostadil was better than that of alprostadil alone [SMD = −0.81, 95%CI (−1.04, −0.58), *P* < .00001] (Fig. 9).

#### 3.5.3. Meta-analysis of urinary protein index

1. 24-hour urinary protein quantification

**Figure 10.**
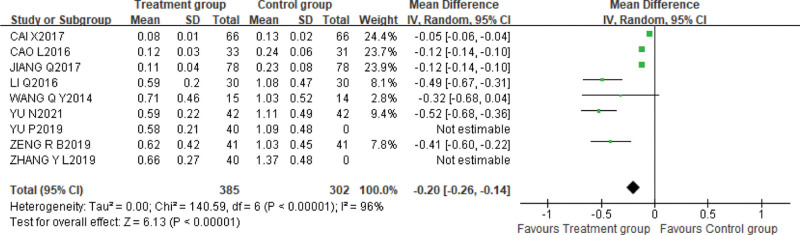
The results of the meta-analysis showed that the treatment group was significantly better than the control group in reducing the level of 24-hour urinary protein quantification [MD = −0.20, 95%CI (−0.26, −0.14), *P* < .00001].

Nine studies^[9,10,13–15,17–20]^ used 24hUp as an outcome measure, with 385 cases in the treatment group and 302 cases in the control group. The data were evaluated using the random effects model due to the high heterogeneity (*P* < .00001, *I^2^* = 96%), and the results of meta-analysis showed that the treatment group was significantly better than the control group in reducing the level of 24hUp [MD = −0.20, 95%CI (−0.26, −0.14), *P* < .00001] (Fig. 10).

2. Urinary albumin excretion rate

**Figure 11.**
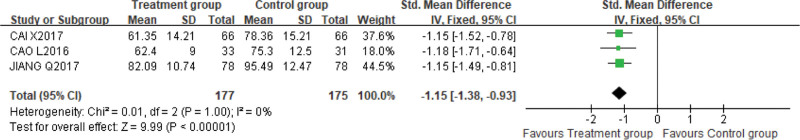
The results of the meta-analysis showed that the treatment group was superior to the control group in reducing the level of Urinary albumin excretion rate [SMD = −1.15, 95% CI (−1.38, −0.93), *P* < .00001].

Three studies^[13,18,19]^ used UAER as the outcome index. Due to the inconsistency of units between studies, SMD was used for analysis. The fixed effect models were used due to lower heterogeneity (*P* = 1.00, *I^2^* = 0%). The results of meta-analysis showed that the treatment group was superior to the control group in reducing the level of UAER [SMD = −1.15, 95% CI (−1.38, −0.93), *P* < .00001] (Fig. 11).

#### 3.5.4. Meta-analysis of adverse reactions

A study reported the occurrence of adverse reactions. Cai Fanliang^[11]^ found that there were 2 cases of regional skin pruritus, one case of headache and 2 cases of fatigue in XBJI combined with alprostadil group, and the incidence of adverse reactions was 16.13%. There was one case of headache and 2 cases of fatigue in the alprostadil group, and the incidence of adverse reactions was 9.68%. There was no significant difference between the 2 groups (*P* > .05). Other studies did not report the safety of XBJI.

### 3.6. Publication bias and sensitivity analysis

**Figure 12.**
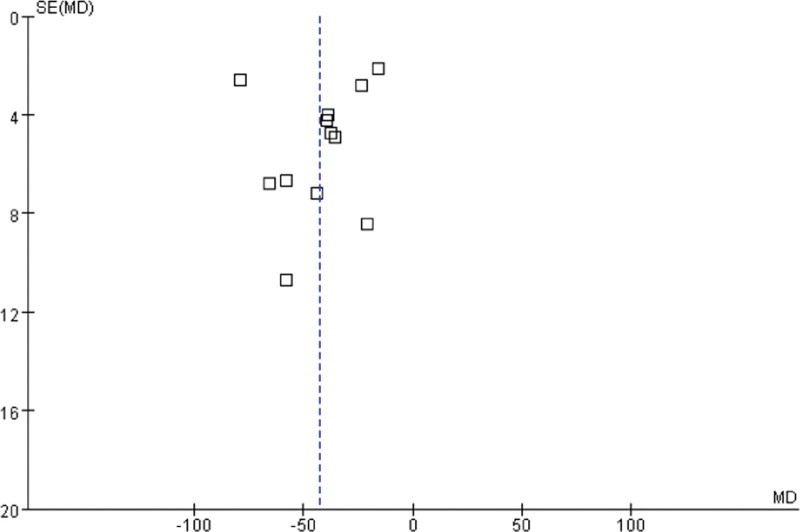
The funnel chart was drawn by Revman 5.3 software to detect the publication bias of outcome indicators (Scr and BUN). As shown in the figure, the funnel diagram of serum creatinine and blood urea nitrogen has poor left-right symmetry, suggesting that there may be a risk of publication bias.

**Figure 13.**
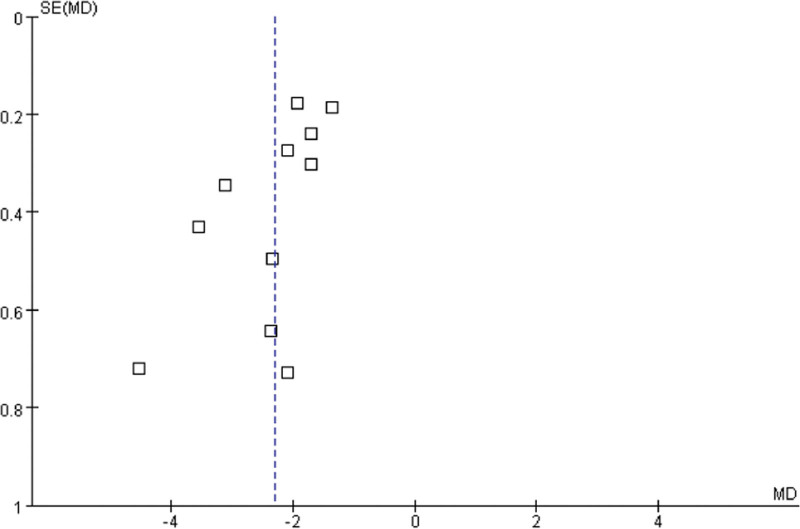
The funnel chart was drawn by Revman 5.3 software to detect the publication bias of outcome indicators (Scr and BUN). As shown in the figure, the funnel diagram of serum creatinine and blood urea nitrogen has poor left-right symmetry, suggesting that there may be a risk of publication bias.

The funnel chart was drawn by Revman 5.3 software to detect the publication bias of outcome indicators (Scr and BUN). As shown in the figure, the funnel diagram of Scr and BUN has poor left-right symmetry, suggesting that there may be a risk of publication bias (Figs. 12 and 13). The sensitivity analysis was used to exclude each study one-by-one and conduct meta-analysis again, it was found that when the study of Jiang Qiang^[13]^ was excluded, the heterogeneity of the outcome index FPG decreased significantly (*P* = .80, *I^2^* = 0%). Further research found that the number of people included in the study of Jiang Qiang^[13]^ was higher than that of other studies. Of course, this heterogeneity may also be related to the baseline level of the included population and the difference in the treatment cycle between groups. The heterogeneity of other outcome indicators did not change significantly compared with the previous results, indicating that the results of meta-analysis were stable, so it proved that the results of this study were reliable.

## 4. Discussion

### 4.1. Blood stasis is the key pathogenesis of diabetic nephropathy

DN is one of the complications of diabetes microangiopathy with proteinuria and progressive renal dysfunction as the primary clinical manifestations. At present, it has become the main cause of end-stage renal disease and renal replacement therapy.^[23]^ DN can be classified into the categories of “Guan Ge,” “Niao Zhuo” and “Shen Xiao” in traditional Chinese medicine.^[24]^ At present, most doctors believe that the pathogenesis of DN belongs to the deficiency of the spleen and kidney and the deficiency of Qi and Yin. The deficiency of the spleen and kidney is primarily responsible for phlegm turbidity and blood stasis, of which blood stasis is its core pathogenesis.^[25]^ Blood stasis runs through DN and affects its occurrence and development. It is not only the pathological product of DN, but also the pathogenic factor of DN.^[26]^

Traditional Chinese medicine believes that the kidney is the place where collaterals gather. Due to the deficiency of spleen and kidney qi and Yin, the spleen fails to function appropriately, and the kidney fails to open and close, resulting in the accumulation of phlegm and blood stasis. Phlegm dampness and blood stasis are effortless to block the operation of Qi and blood. The disease will affect the kidney over time, resulting in the stagnation of kidney collaterals and blood stasis. Later, Professor Lv Renhe^[27]^ put forward the theory of “Micro syndrome of renal collaterals.” The professor believes that with the development of DN, pathological evil Qi such as Qi stagnation, blood stasis and phlegm dampness appear in the body. The evil Qi turns into heat after depression for a long time, and the blood is burned to produce blood stasis, which eventually leads to renal collateral block. Professor Zhang Daning,^[28]^ master of national physician, believes that the main pathogenesis of DN is attributed to “kidney deficiency and blood stasis.” Kidney deficiency and blood stasis are interrelated and do not exist in isolation. Kidney deficiency must be blended with blood stasis. Blood stasis aggravates kidney deficiency at the same time, and the 2 affect each other.

Modern medical research has identified that DN patients are frequently accompanied by abnormal activation of immune inflammation, disorder of lipid metabolism, hemorheological changes and microcirculation disorders, which further verify the existence of blood stasis from the perspective of modern medicine.^[29]^ It can be demonstrated that blood stasis runs through the development of DN and becomes the crucial pathogenesis of DN.

### 4.2. Xuebijing injection fits the pathogenesis of diabetic nephropathy blood stasis syndrome

XBJI is extracted from Hong Hua, Chi Shao, Dang Gui, Chuan Xiong, Dan Shenand other effective components of traditional Chinese medicine.

Hong Hua has the effects of dredging meridians, relieving discomfort, encouraging blood circulation and removing blood stasis. Relevant studies demonstrate that^[30]^ safflower yellow pigment and safflower quinone glycoside can effectively shorten the length of thrombus in mice with blood stasis syndrome, prolong the time of thrombin and prothrombin, enhance the fibrinolytic activity, and perform an anticoagulant and antithrombotic role.

Chi Shao can clear heat and cool blood, promote blood circulation, and disperse blood stasis. The study found that^[31]^ pavilion and paeoniflorin contained in Chi Shao can reduce the activity of coagulation factors, improve the aggregation of red blood cells and platelets, increase the content of nitric oxide, promote vasodilation and prevent thrombosis.

Dang Gui and Chuan Xiong are classic drug pairs that replenish blood and activate blood without harm blood^[32]^ and their common component ferulic acid can prolong prothrombin time and inhibit thrombosis by inhibiting the release of thromboxane and serotonin, the activation and aggregation of platelets.

Dan Shen has the effects of promoting blood circulation and dredging meridians, dispersing blood stasis and relieving discomfort. Relevant studies demonstrate that^[33]^ Dan Shen can enhance renal blood flow, enhance microcirculation, increase glomerular filtration rate, and then delay glomerular injury by widening coronary artery. Consequently, in general, the drugs in XBJI play a role in encouraging blood circulation and eliminating blood stasis, which is in line with the pathogenesis of DN blood stasis syndrome.

### 4.3. Limitations

As we have done, this is the first meta-analysis reported on all RCTs of XBJI combined with alprostadil in the treatment of DN. The final meta-analysis results verified that XBJI combined with alprostadil can better decrease blood glucose and blood lipid, improve renal function and reduce proteinuria in patients with DN. However, there are still some deficiencies: the language is limited to Chinese, and there is a high risk of bias in the included population; Only 7 studies^[9,11,13,15,18,19,22]^ in the included literature described the specific random allocation method, but 14 studies^[9–22]^ did not mention the allocation concealment method and the implementation of blind method; Only one study^[11]^ reported adverse reactions, so it is impossible to comprehensively evaluate the safety of XBJI combined with alprostadil; Some indicators are too low for the included population, resulting in insufficient sample size; DN is a chronic disease. When assessing the efficacy of XBJI combined with alprostadil, its long-term therapeutic effect should be considered. Unfortunately, the course of treatment is primarily 1 to 2 weeks in the incorporated studies, so it is impossible to know its long-term impact on DN patients. The diagnostic criteria are not unified: 4 studies^[15,17,20,21]^ made clear that the diagnostic basis of DN refers to “the diagnostic, syndrome differentiation and efficacy evaluation criteria of diabetes nephropathy,” one study^[18]^ made reference to “internal medicine,” and the rest did not make clear the diagnostic basis of DN; Different stages of DN: the DN population included in the 2 studies^[19,20]^ met the stage IV diagnostic criteria of Mogensen stage, and the rest only indicated that the included population was DN patients; Some studies used inappropriate randomization: 3 studies^[10,14,17]^ were grouped according to “different treatment methods,” considering that there was a great risk of bias.

## 5. Conclusion

Meta-analysis demonstrated that XBJI combined with alprostadil could more effectively enhance the blood glucose, blood lipid, renal function, urinary protein and other related indicators of DN patients, improve the quality of life of patients and delay the progress of DN.

In terms of adverse reactions, only one study^[11]^ reported adverse reactions such as skin pruritus, headache and fatigue during the treatment, but the number was small, and the recent meta-analysis showed that,^[34]^ XBJI can improve the efficacy of blood purification in the treatment of systemic inflammatory response syndrome, inhibit inflammatory reaction, and will not increase the incidence of adverse reactions.

However, relevant studies also found that^[35]^ when the concentration of XBJI is higher than the clinical recommended concentration, or the drug is stored for too long and the injection speed is too fast, it will aggravate the degree of allergic reaction in model mice. At the same time, XBJI contains multiple sensitizing components such as protein, pigment, resin and volatile oil, which may be the source of adverse reactions.^[36]^ Therefore, the use of XBJI should be carried out in strict accordance with the usage, dose, preparation concentration and infusion speed recommended in the corresponding drug operation manual, and follow the principle of current use and preparation, which will be of positive significance to improve the drug safety of XBJI. At the same time, a large sample, higher research quality, multi center and more rigorous RCTs are needed in the future, in order to evaluate the efficacy of XBJI combined with alprostadil in the treatment of DN more accurately and objectively.

## Author contributions

**Methodology:** Na Hao, Yang Liu.

**Software:** Yang Liu, Luxuan Guo, Pengbo Zhao.

**Writing – original draft:** Yang Liu, Wanwen Li.

**Writing – review & editing:** Na Hao.
